# Quantum bath engineering of a high impedance microwave mode through quasiparticle tunneling

**DOI:** 10.1038/s41467-022-34762-z

**Published:** 2022-11-21

**Authors:** Gianluca Aiello, Mathieu Féchant, Alexis Morvan, Julien Basset, Marco Aprili, Julien Gabelli, Jérôme Estève

**Affiliations:** Laboratoire de Physique des Solides, CNRS, Université Paris Saclay, Orsay, France

**Keywords:** Single photons and quantum effects, Superconducting devices

## Abstract

In microwave quantum optics, dissipation usually corresponds to quantum jumps, where photons are lost one by one. Here we demonstrate a new approach to dissipation engineering. By coupling a high impedance microwave resonator to a tunnel junction, we use the photoassisted tunneling of quasiparticles as a tunable dissipative process. We are able to adjust the minimum number of lost photons per tunneling event to be one, two or more, through a dc voltage. Consequently, different Fock states of the resonator experience different loss processes. Causality then implies that each state experiences a different energy (Lamb) shift, as confirmed experimentally. This photoassisted tunneling process is analogous to a photoelectric effect, which requires a quantum description of light to be quantitatively understood. This work opens up new possibilities for quantum state manipulation in superconducting circuits, which do not rely on the Josephson effect.

## Abstract (web summary)

Quantum bath engineering in the context of circuit quantum electrodynamics typically relies on single-photon losses. Aiello et al. demonstrate an approach for engineering higher-order photon losses in a microwave resonator coupled to a tunnel junction, which may be utilized in quantum information applications.

## Introduction

Quantum bath engineering is considered as a promising route to perform certain tasks in quantum information processing, such as state stabilization, passive error correction, or fast qubit initialization^1–8^. In the context of circuit QED, bath engineering usually results from the interplay between coherent evolution and dissipation in the form of single-photon loss^9^. Such engineered losses, in particular two-photon losses, are at the heart of promising error correction schemes in superconducting qubit architectures^8^. Here, we demonstrate a different approach where engineered dissipation comes from the non-linear coupling of a microwave mode to a tunnel junction, which realizes a bath consisting of two electronic reservoirs. Dissipation arises from the photoassisted tunneling processes, during which one electron tunnels, while *l* photons are absorbed from the mode. Because the mode is sustained by a high kinetic inductance superconducting resonator made of granular Aluminum, its characteristic impedance is sufficiently large such that the high order loss processes with *l* > 1 are allowed^10–15^. The rate of processes with given *l* can be tuned through the dc voltage that biases the junction. As an example of engineered dissipation, we focus on the regime where *l* ≥ 2 processes dominate over single photon loss. The dynamics is then restricted by the quantum Zeno effect to the subspace spanned by the zero and one photon Fock states^16^, turning the harmonic oscillator mode into a two-level system.

From a broader perspective, photoassisted tunneling is a special case of photoelectric effect, where the electron is emitted into contact rather than in free space. As with the photoelectric effect, the frequency of the light must exceed the chemical potential difference between the two contacts divided by the Planck constant in order to observe photoassisted tunneling at low light intensity. The natural interpretation for this threshold behavior uses the concept of photon as discussed above. But, in most cases, the electric field that is responsible for the electron emission may be considered as a classical field. Even though the photoelectric effect leads Einstein to propose the idea of photon, the standard semiclassical model of light–matter interaction, which neglects the quantum nature of light and treats only matter at the quantum level, accurately describes all photoemission experiments^17^. This paradox has been known and debated for a long time^18^. Our experiment sheds new light on this problem by reaching a regime, where both matter and light must be treated at the quantum level in order to reach a quantitative understanding. In the context of a microwave resonator coupled to a tunnel junction, the semiclassical approach describes the junction in terms of admittance, which can then be used to model its effect on the resonator mode coupled to the junction^19,20^. In the last part of the paper, we compare the predictions of this model to the ones of the full quantum model, in particular for the frequency shift of the resonator. Our data confirm that quantum effects significantly contribute to the induced energy shift, the so-called Lamb shift^21^. Furthermore, the energy shift is different for each Fock state, which is meaningless in a classical model.

## Results

**Fig. 1:**
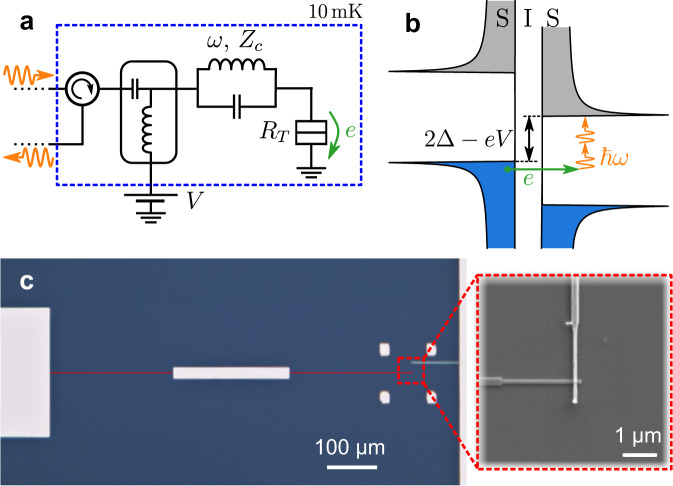
Experiment principles. **a** Schematic of the experimental circuit. A microwave mode at frequency *ω* ≈ 2*π* × 6 GHz, here represented by an *LC* resonator, is coupled to a superconducting tunnel junction with tunnel resistance *R*_*T*_. The characteristic impedance *Z*_*c*_ of the mode is 4.5 kΩ, much larger than in a conventional superconducting resonator. A bias tee and a circulator are used to dc bias the sample while measuring the reflected microwave signal. **b** When the bias voltage is such that 2Δ − *eV* > *lℏω*, the tunneling of quasiparticles through the junction is allowed only if at least *l* photons (here two) are absorbed from the mode to provide the missing energy. **c** Microscope image of the sample realizing the circuit shown in **a**. The resonator consists of two grAl quarter wave resonators (red) connected by a wider Al wire (white). The tunnel junction (Al/AlO_*x*_/Al) connects the resonator right end to the ground. The junction area is 150 × 150 nm^2^, leading to a tunnel resistance *R*_*T*_ = 150 kΩ far above the gap. A microstrip Al line connects the resonator’s left end to the measurement circuit. All experiments are performed in a dilution fridge with a base temperature of 10 mK.

The principle of the experiment is presented in Fig. 1. A high impedance resonator with a resonant mode around *ω* ≈ 2*π* × 6 GHz is galvanically coupled to a tunnel junction as schematically depicted in Fig. 1a. In addition to the usual single-photon loss, due to the coupling to the measurement line or to intrinsic loss mechanisms, photons in the mode may also be absorbed through the photoassisted tunneling of a quasiparticle across the junction (Fig. 1b)^19,22^. Such inelastic tunneling processes, where *l* photons are absorbed, are energetically allowed only if the bias voltage *V* is sufficiently close to the gap, *eV* > 2Δ − *lℏω*, where *Δ* ≈ 200 μeV is the superconducting gap in each Al electrode. The junction thus realizes a tunable quantum absorber, where the minimum number of absorbed photons per photoassisted tunneling event is set by the voltage bias.

In order for the engineered loss to be efficient, the corresponding rate must dominate single photon loss, which is only possible if the characteristic impedance of the mode coupled to the junction is sufficiently large. The expected rate for the photoassisted tunneling process can be derived from the tunnel Hamiltonian, which describes the coupling between the tunneling electrons and an electromagnetic mode^23^

1 $${\hat{H}}_{{{{{{{{\rm{T}}}}}}}}}={e}^{i\lambda (\hat{a}+{\hat{a}}^{{{{\dagger}}} })}\, \hat{B}+{{{{{{{\rm{h.c.}}}}}}}}\,.$$

The operator $\hat{a}$ is the annihilation operator of the considered mode and $\hat{B}=T{\sum }_{LR}{\hat{c}}_{R}^{{{{\dagger}}} }{\hat{c}}_{L}$ transfers one electron from the left (*L*) to the right (*R*) junction contact. The barrier transparency *T* is inversely proportional to the junction resistance *R*_*T*_ at voltages far above the gap. The displacement operator ${e}^{i\lambda (\hat{a}+{\hat{a}}^{{{{\dagger}}} })}$ can be interpreted as a consequence of charge conservation: one tunneling event corresponds to a translation of the charge degree of freedom by one electron^23^. The displacement amplitude, *λ*, is proportional to the zero point fluctuations of the conjugate of the charge operator and is given by $\lambda=\sqrt{\pi {Z}_{c}/{R}_{K}}$, where *Z*_*c*_ is the characteristic impedance of the mode and *R*_*K*_ = *h*/*e*^2^ is the quantum of resistance. This is similar to the displacement operator that appears in the coupling Hamiltonian between light and an atom trapped in an harmonic potential as a consequence of momentum conservation^24^.

The matrix elements of the displacement operator between two Fock states $\left|n\right\rangle$ and $\left|n+l\right\rangle$ with *l* ≥ 0 are the Franck-Condon coefficients, which depend on *λ* as^10,25,26^

$${\alpha }_{nl}=|\langle n+l|e^{i\lambda (\hat{a}+{\hat{a}}^{{{{\dagger}}} })}|n\rangle|^{2}=\frac{{\lambda }^{2l}{e}^{-{\lambda }^{2}}n!}{(n+l)!}{L}_{n}^{(l)}{({\lambda }^{2})}^{2}\,,$$

where ${L}_{n}^{(l)}$ are the generalized Laguerre polynomials. In a standard superconducting resonator, *Z*_*c*_ is much smaller than *R*_*K*_, resulting in *λ* ≪ 1, which is analog to the Lamb–Dicke regime for atoms^24^. In this case, processes between Fock states differing by *l* are exponentially suppressed as *λ*^2*l*^. Here, we are interested in the opposite regime, where *λ* ~ 1. In this case, the displacement amplitude in the mode quadrature phase space is comparable to the extension of the ground state and transitions between different Fock states are allowed.

Considering the left and right contacts as electronic reservoirs at thermal equilibrium, the Fermi golden rule predicts that photoassisted tunneling leads to a loss rate for the $\left|n\right\rangle$ state given by^10–12,25,27^

2 $${\gamma }_{n}=\frac{1}{e}\mathop{\sum }\limits_{l=1}^{n}{\alpha }_{n-l,l}\, I(V+l\hslash \omega /e)\,,$$

where each term in the sum corresponds to the contribution of the *l* photon absorption process. The rate of such a process is proportional to the corresponding Franck-Condon factor multiplied by the current *I* that would flow through the junction in the absence of resonator. Energy conservation implies that the current must be evaluated at the voltage corresponding to the bias voltage shifted by the energy of the *l* photons. Because of the superconducting gap, the *l* photon process is allowed only when *eV* + *lℏω* ≥ 2Δ, otherwise *I* = 0. Note that *I*(*V*) coincides with the actual current flowing through the junction only when photoassisted processes are negligible, i.e. at large voltages above the gap.

### Experimental device and resonator spectroscopy

**Fig. 2:**
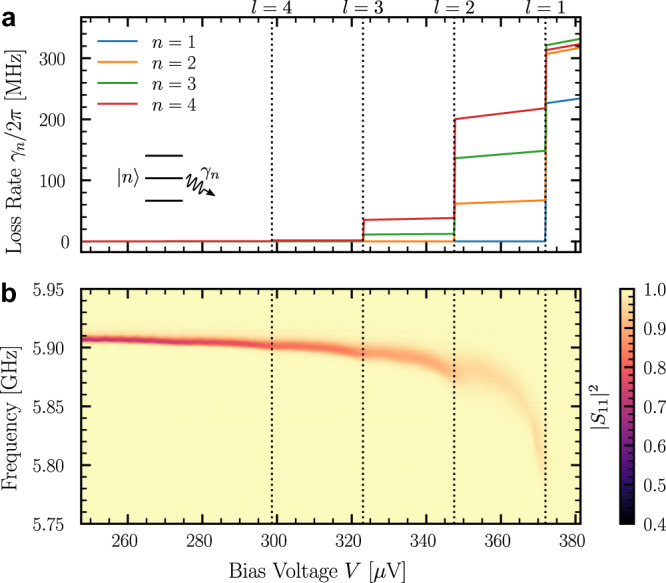
Tunnel junction as a tunable quantum absorber. **a** Quasiparticle tunneling induces an extra loss *γ*_*n*_ (Eq. (2)), which is different for each Fock state of the mode coupled to the junction. Dashed vertical lines show the onset of the *l* photon absorption process, given by (2Δ − *lℏω*)/*e*. The photon number *n* corresponding to the lowest Fock state with increased loss can be chosen with the bias voltage. The parameters correspond to the ones expected in the experiment. **b** Experimental spectroscopy of the 6 GHz mode as a function of the bias voltage *V*. The image shows the measured reflection coefficient ∣*S*_11_∣^2^ for an incident power of −115 dBm on the resonator. The onset of the different absorption processes is clearly visible up to *l* = 4.

In the experiment presented here, we reach *Z*_*c*_ = 4.5 kΩ, which corresponds to *λ* = 0.74. In this regime, high-order processes have Franck-Condon coefficients that are comparable to one of the one photon processes, e.g. *α*_02_ ≈ *α*_01_/3 ≈ 0.1. Figure 2a shows the evolution of *γ*_*n*_ as a function of voltage for parameters corresponding to our experiment. The *I*(*V*) characteristic of the junction is calculated from the resistance *R*_*T*_ = 150 kΩ measured far above the gap. But, in order to take into account the presence of other modes in the resonator, we replace *R*_*T*_ by an effective tunnel resistance with a larger value ${\tilde{R}}_{T}=430\,{{{{{{{\rm{k}}}}}}}}\Omega$. The increase of resistance is given by the product of the dynamical Coulomb blockade factors ${{{\Pi }}}_{n\ne 1}{e}^{{\lambda }_{n}^{2}}$ over all the modes except the one at 6 GHz (see SI). Figure 2a shows that the junction is expected to act as a tunable absorber that can distinguish between the first Fock states up to *n* = 3.

In order to reach *λ* ~ 1, we use granular Aluminum (grAl) as the material of the resonator. GrAl has been shown to be a promising material to realize a superinductance with small loss^28–31^. Other possible methods include resonators with carefully designed geometries^32,33^, the use of other high kinetic inductance superconductors^34–36^ or chains of Josephson junctions^37–40^. To first approximation, the mode probed in the experiment is the fundamental mode of a quarter wavelength resonator made of a 0.5 μm wide and 200 μm long grAl wire with a kinetic inductance of 0.56 nH/□ (see Fig. 1c). The junction connects the end of the resonator, where the mode has a voltage maximum, to the ground. In order to obtain a high-quality factor, despite the galvanic connection to the measurement line, a distributed Bragg reflector (DBR) is inserted between the resonator and the line.

The resonant frequencies, characteristic impedances, and coupling loss rates of the different modes sustained by the structure are numerically simulated (see SI). The design is chosen to obtain a mode with a large characteristic impedance, a resonance frequency close to 6 GHz and a quality factor above 10^4^. The sample is fabricated through standard e-beam lithography and double-angle evaporation (see SI). The exact value of *λ* depends on the precise value of the kinetic inductance of the wire and the junction capacitance, which can only be estimated at the design stage. We deduce the precise values of these two parameters by comparing the simulation to the measured resonance frequencies of the 6 GHz mode as well as the ones of the other modes at 1.9, 12, 24, and 32 GHz. We finally obtain *Z*_*c*_ = 4.5 kΩ for the 6 GHz mode and a quality factor of 1.3 × 10^4^, which corresponds to a coupling loss rate to the measurement line *κ*_*c*_ = 2*π* × 0.45 MHz.

Figure 2b shows the spectroscopy of the 6 GHz mode as a function of the voltage bias close to the superconducting gap 2Δ/*e*. The incoming microwave power is chosen to populate many Fock states so that we can observe the onset of the different loss processes every time *eV* > 2Δ − *lℏω* (vertical dashed lines). When a new loss process is allowed, the intensity in the mode decreases, leading to a diminution of the reflection dip. At the same time, the width of the resonance increases. When the one-photon absorption process becomes allowed, the resonance abruptly disappears. This is because the corresponding loss rate, which affects every Fock states, is much larger than *κ*_*c*_ (see Fig. 2a). The mode becomes under-coupled and the reflection dip vanishes. At the same time that loss increases as the voltage increases, the resonance frequency redshifts as a consequence of the Kramers–Kronig relations. In particular, we observe small frequency kinks every time a new loss process appears. These frequency shifts will be detailed at the end of the manuscript. At lower voltages (not shown here), we observe multiple kinks in the spectrum that correspond to inelastic Cooper pair tunneling resonances. These results and their analysis will be presented elsewhere.

### Quantum Zeno dynamics

**Fig. 3:**
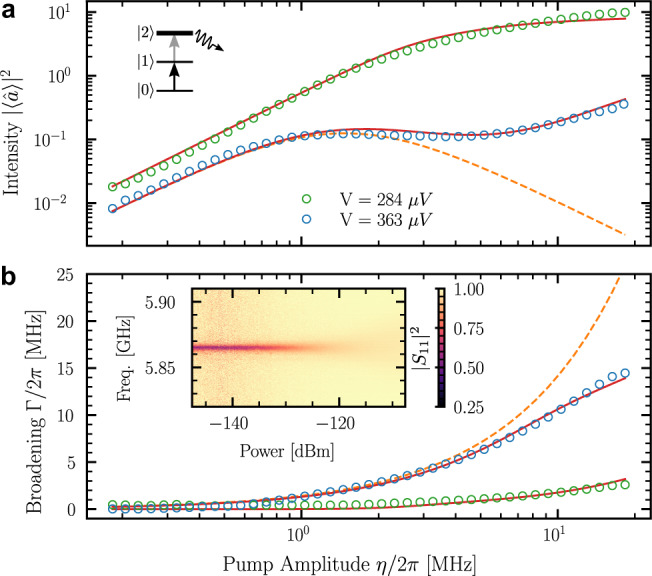
Quantum Zeno dynamics. **a** Evolution of the squared mean amplitude ∣〈*â*〉∣^2^ in the mode as a function of the pump amplitude *η* for two different bias voltages. In the absence of Zeno effect, ∣〈*â*〉∣^2^ is quadratic with the pump amplitude (green data). When the voltage lies in the range where the quantum Zeno dynamics limits the dynamic to one of a two-level system, we observe a clear saturation (blue data). The solid red line shows the prediction of a master equation taking into account the different absorption rates for different Fock states (see SI). The dashed line shows the expectation for an ideal two-level system. The calibration of the measured intensity is detailed in the SI. **b** Evolution of the power broadening Γ as a function of the pump amplitude. The resonance spectra shown in the inset are fitted using the usual formula for a two-level system, which predicts a fwhm $\sqrt{{\kappa }^{2}+2{{{\Gamma }}}^{2}}$ (see SI), where *κ* is the total loss rate. Power broadening is important in the case of Zeno dynamics (blue data) and negligible otherwise, except at very high power (green data). The solid and dashed lines show the result of the master equation simulation and the expectation for an ideal two-level system.

The step-like increase of the *l* photon loss rate at *eV* = 2Δ − *lℏω* is ideally suited to induce quantum Zeno dynamics and engineer the Hilbert space of the 6 GHz mode. If the voltage is set in the range 2Δ − *ℏω* > *eV* > 2Δ − 2*ℏω*, all Fock states except $\left|0\right\rangle$ and $\left|1\right\rangle$ are lossy because of *l* ≥ 2 processes. In particular, the $\left|2\right\rangle$ state experiences a two-photon loss rate on the order of *γ*_2_ ≈ 2*π* × 65 MHz, while the single photon loss induced by the junction is expected to be negligible. We bias the junction in this voltage range, pump the mode with a microwave tone and measure the reflected signal with an homodyne detector. The intensity in the mode at resonance is shown in Fig. 3a as a function of the pump amplitude *η*, which is related to the incoming pump power *P* as $\eta=\sqrt{{\kappa }_{c}P/\hslash \omega }$. Because of the engineered dissipation, a state initially in the subspace spanned by $\left|0\right\rangle$ and $\left|1\right\rangle$ is continuously projected in this subspace in the absence of tunneling event. This nondestructive measurement induces quantum Zeno dynamics in this subspace and the mode behaves as a two-level system rather than an harmonic oscillator. This restriction of the Hilbert space is efficient as long as *η* remains small compared to the projection rate, which is here set by the photon loss rate *γ*_2_. When this is the case, we observe a saturation of the intensity near ∣〈*â*〉∣^2^ = 1/8, as expected for a two-level system. This is a clear signature of the reduction of the Hilbert space to the $\left|0\right\rangle$ and $\left|1\right\rangle$ subspace. At high pump power, the quantum Zeno effect breaks down and the mode intensity starts to increase again. Because the state experiences large losses in the subspace spanned by the Fock states with *n* ≥ 2, this increase is slower than the one expected for an ideal Zeno effect where the system would simply be projected in the subspace. Numerical simulations indicate that the breakdown of the Zeno happens around *η* = 2π ×3 MHz (see SI). In the two-level saturation regime, we also observe that the resonance width increases because of power broadening (see Fig. 3b). We use this effect in order to calibrate the pump amplitude by assuming that the broadening is linear in pump intensity at low pump power, as with an ideal two-level system. The solid lines in Fig. 3 show the results of the numerical simulation of a master equation describing the evolution of the mode coupled to the junction as detailed in SI^11,12^. Our data are well reproduced by a single-mode model using the expected effective tunnel resistance ${\tilde{R}}_{T}$ and including an additional single-photon loss rate of a few MHz. The simulation includes the Lamb shift of the different levels, which also contributes to the blockade of the 1 → 2 transition (see below). The figure of merit of the observed Zeno blockade can be quantified by the ratio between the effective loss rate from $\left|2\right\rangle$ and from $\left|1\right\rangle$ that reproduces our data, which is around 25 for our experiment.

### Lamb Shift

We now turn to a detailed analysis of the shift of the resonance frequency as a function of the voltage bias. The frequency shift experienced by each Fock state is related to the loss rate via the Kramers–Kronig relations. We introduce the Kramers-Kronig (KK) transform of the current–voltage characteristic as^19^

$${I}^{{{{{{{{\rm{KK}}}}}}}}}(V)=\frac{1}{\pi }{{{{{{{\mathcal{P}}}}}}}}\int\nolimits_{-\infty }^{\infty }\frac{I({V}^{{\prime} })}{{V}^{{\prime} }-V}\,d{V}^{{\prime} }\,.$$

The frequency shift of the $\left|n\right\rangle$ state, which is usually called Lamb-shift^21^, is then given by^12^

3 $$\delta {\omega }_{n}=-\frac{1}{2e}\left(\mathop{\sum }\limits_{l=1}^{n}{\alpha }_{n-l,l}\, {I}_{l}^{{{{{{{{\rm{KK}}}}}}}}}+\mathop{\sum }\limits_{l=0}^{\infty }{\alpha }_{n,l}\, {I}_{-l}^{{{{{{{{\rm{KK}}}}}}}}}\right)\,,$$

where ${I}_{l}^{{{{{{{{\rm{KK}}}}}}}}}$ stands for *I*^KK^(*V* + *lℏω*/*e*). The first term corresponds to the KK transform of (2). The second term is absent from (2), because the corresponding rates vanish for voltages below the gap, which is not the case for the KK transform, i.e. *I*^KK^(*V*) is not zero when *eV* < 2Δ (see SI for a plot of *I*^KK^(*V*)). From (3), the shift of the resonance frequency for the fundamental 0 → 1 transition is given by

4 $$\delta {\omega }_{01}=-\frac{{\lambda }^{2}{e}^{-{\lambda }^{2}}}{2e}\mathop{\sum }\limits_{l=0}^{\infty }\frac{{\lambda }^{2l}}{l!}\left({I}_{-l-1}^{{{{{{{{\rm{KK}}}}}}}}}+{I}_{-l+1}^{{{{{{{{\rm{KK}}}}}}}}}-2{I}_{-l}^{{{{{{{{\rm{KK}}}}}}}}}\right)$$

Keeping only the *l* = 0 term and neglecting the ${e}^{-{\lambda }^{2}}$ term leads to the frequency shift that is derived from a classical treatment of the electromagnetic field^19^, in which case the reactive part of the junction admittance at low intensity can be written as $e({I}_{1}^{{{{{{{{\rm{KK}}}}}}}}}+{I}_{-1}^{{{{{{{{\rm{KK}}}}}}}}}-2{I}_{0}^{{{{{{{{\rm{KK}}}}}}}}})/(2\hslash \omega )$ as measured in refs. 13, 20, 41. This classical expression also corresponds to the first term of the Taylor expansion of (4) in powers of *λ*. In our case, because *λ* is large, this approximation is not valid and the higher-order terms are expected to significantly contribute to the frequency shift.

**Fig. 4:**
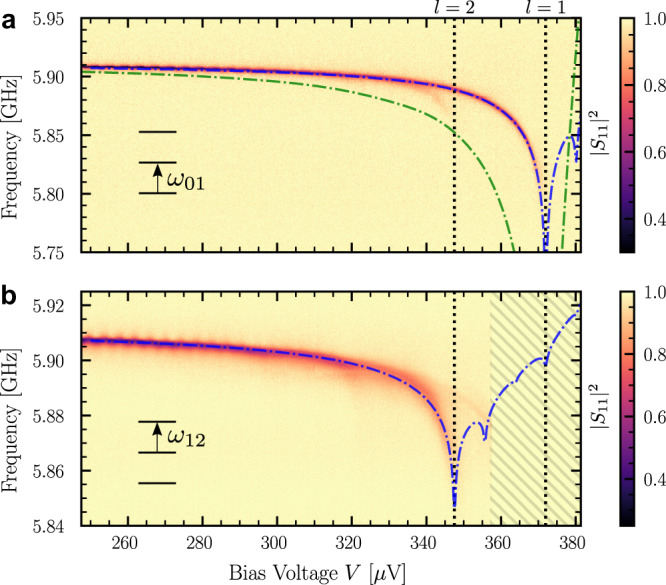
One and two-photon spectroscopy as a function of voltage. **a** Reflected signal measured with an injected microwave power of −140 dBm (*η* ≈ 2*π* × 2 MHz) as a function of frequency and bias voltage. The power is sufficiently low to mostly probe the 0 → 1 transition. The vertical lines are the same as in Fig. 1. **b** Reflected signal in the presence of a second microwave tone tuned at the frequency measured in **a**. This two-photon spectroscopy probes the 1 → 2 transition, which shifts differently than the 0 → 1 transition, showing the non-linearity induced by the Lamb shift. In both cases, the dashed blue lines correspond to the prediction of an ab initio quantum model. The dashed green line in **a** corresponds to the classical approximation, keeping only terms of order *λ*^2^ in the expression of the frequency shift. The hatched area was not measured.

Figure 4a shows the reflection spectrum that we measure at very low pump power in order to probe the 0 → 1 transition only. We compare it to the predictions of the classical admittance model (dashed green line) and to the one of the quantum model (dashed blue line). Because of the presence of other modes in the resonator than the 6 GHz mode, the expression for *δω*_*n*_ that we use in the quantum description is slightly more involved than the one given in (3) and is given in the SI. As expected, the two models significantly differ and our data are in good agreement with the ab initio quantum model. This is a rare situation, similar to the original Lamb shift effect^42^, where quantum effects significantly affect the frequency shift. The *λ* coefficient may be rewritten as *λ*^2^ = 2*παZ*_*c*_/*Z*_vac_, where *α* ≈ 1/137 is the fine structure constant and *Z*_vac_ ≈ 377 Ω is the impedance of free space, showing that the expansion (4) in powers of *λ* is actually an expansion in powers of *α* as expected for a QED effect. A similar result was obtained in the dual situation where a transmon qubit is frequency shifted by a high impedance environment^40^. Equation (4) shows that the quantum correction is more than a simple renormalization of the resistance by the factor ${e}^{{\lambda }^{2}}$.

Equation (3) also predicts that the Lamb shift terms introduce a non-linearity in the harmonic spectrum of the mode as a consequence of the non-linear bath coupling. This effect is already visible in Figs. 2b and 4a, where we observe a shift and even a splitting of the resonance when *eV* ≈ 2Δ − *lℏω*. In order to confirm that this splitting can be attributed to different shifts of the different Fock states, we perform a two-photon spectroscopy as shown in Fig. 4b. A first tone is tuned to excite the 0 → 1 resonance that we measure at very low power (Fig. 4a). We then acquire a reflection spectrum using a second tone that mostly probes the 1 → 2 transition. The signal is only visible when *eV* ≤ 2Δ − 2*ℏω* for the same reason as in Fig. 4a. We clearly observe a frequency shift of the 1 → 2 transition, which is different from the one of the 0 → 1 transition, in very good agreement with the quantum model. This non-linear effect favors the observed restriction of the Hilbert space to the first two levels. The induced non-linearity is maximum around 348 *μ*V and equal to (*ω*_12_ − *ω*_01_)/2*π* ≃ 42 MHz. The nonlinear shifts due to the *l* > 2 terms in (3) are responsible for the kinks in the resonance frequency at *eV* = 2Δ − *lℏω* observed in Fig. 2b.

In conclusion, we have demonstrated a new way to engineer dissipation in superconducting QED circuits by taking advantage of the nonlinear coupling between a high-impedance mode and electronic reservoirs. The dominant loss mechanism can be tuned to be a one, two, or even higher-order photon process. Our results could be extended to other types of junctions with a nonlinear current voltage characteristic. Such engineered dissipation could have applications in quantum computing for rapid initialization of a microwave mode to vacuum, or to stabilize states in error correction schemes. More fundamentally, our results give an example of a situation where quantum effects invalidate the classical approach to dissipation based on linear response theory, for example in terms of admittance, to describe the coupling between the different elements of a circuit.

## Supplementary information

Supplementary Information

Peer Review File

## Data Availability

The data that support the findings of this study may be made available from the corresponding authors upon reasonable request.

## Code availability

The code used for the analyses may be made available from the corresponding authors upon reasonable request.
