# Supplementary material for: Quantum bath engineering of a high impedance microwave mode through quasiparticle tunneling
Source: Nat Commun. 2022 Nov 21;13:7146. doi: 10.1038/s41467-022-34762-z (PMC9681747; doi:10.1038/s41467-022-34762-z)
Supplement: Supplementary file 1 — Supplementary Information [file 41467_2022_34762_MOESM1_ESM.pdf]

Supplementary Information
Quantum bath engineering of a high impedance microwave mode through quasiparticle tunneling

Gianluca Aiello[1], Mathieu Féchant[1], Alexis Morvan[1], Julien Basset[1], Marco Aprili[1], Julien Gabelli[1], and Jérôme Estève[1]
[1]*Laboratoire de Physique des Solides, CNRS, Université Paris Saclay, Orsay, France*

I. SAMPLE GEOMETRY AND EM SIMULATIONS

The resonator consists in a quarter wavelength microstrip line, which is galvanically coupled to a 50 Ω line through a distributed Bragg reflector (DBR). In order to obtain high impedance modes, the resonator is made of granular Aluminum (grAl), which has a high kinetic inductance, and the width of the microstrip line is reduced to 0.5 µm. Our grAl layer has a typical sheet resistance of 0.8 kΩ/□ (see below for fabrication details), which corresponds to a sheet inductance of 0.7 nH/□, assuming a critical temperature of 1.85 K. With these parameters, simulations using the Sonnet software indicate that the impedance of the line is 6 kΩ on a standard Silicon wafer. Note that this value may slightly differ from one sample to the other because of fluctuations in the grAl composition, which changes both the resistivity and the critical temperature.

The DBR consists in two sections of microstrip lines with a large impedance mismatch. The first high impedance section is identical to the resonator, while the second section is made of Aluminum and has a much lower impedance of 105 Ω. The impedance mismatch between the two sections comes both, from the use of different materials, one with a large kinetic inductance and one without, and from the width difference, which is 0.5 µm for the high impedance section and 20 µm for the low impedance section.

In the final design of the DBR, we reduce on purpose the length of the second (low impedance) section below the quarter wavelength Bragg condition. Otherwise, the quality factor of the fundamental mode of the resonator would be too high. The length is adjusted to target a quality factor of approximately 1.3×10^4 Finally, an Al/AlOx/Al junction connects the left end of the resonator to the ground. A schematics of the sample is shown in figure S1.

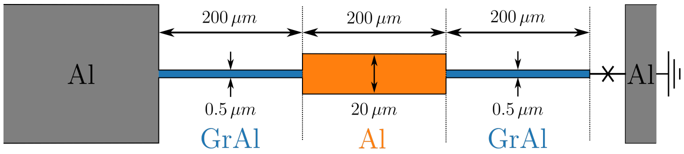

FIG. S1: Geometry of the sample. The resonator and the first DBR section are made of GrAl and have a length that corresponds to one quarter wavelength at 6 GHz. The second section is made of Al and has a much lower impedance. The junction connects the end of the resonator to the ground.

In order to evaluate the properties of the resonant modes of the system, we simulate the sample using the Sonnet software. The resonance frequency and the characteristic impedance of each mode are calculated from the admittance $Y(\omega)$ seen by the junction. We first numerically simulate the admittance matrix $Y_{ij}(\omega)$ of the two port network that is obtained by placing a first port at the position of the junction and a second one on the 50 Ω measurement line. From the admittance matrix, we then compute the admittance seen by the junction by eliminating the second port and taking into account the capacitance of the junction C_J. We arrive at

$$Y(\omega) = Y_{11} - \frac{Y_{12}Y_{21}}{Y_{22} + Y_0} + i\omega C_J \,, \tag{S.1}$$

where $Y_0 = 1/50\ \Omega^{-1}$ is the admittance of the measurement line.

The resonant frequencies ω_n of the modes are obtained by looking for the zeros of $\mathrm{Im}[Y(\omega)]$, while their characteristic impedance is given by [1]

$$Z_n = \frac{2i}{\omega_n}\left(\frac{\partial Y(\omega)}{\partial \omega}\Big|_{\omega=\omega_n}\right)^{-1} . \tag{S.2}$$

In order to estimate C_J and the precise value of the grAl sheet inductance, we compare the measured frequencies of the modes to the simulated ones. We find that a value of $C_J = 1.75$ fF and a sheet inductance of 0.56 nH/□ explains

well our results. It is compatible with the 150×150 nm^2 area of our junction. The measured values of the resonance frequencies are obtained from photo-assisted current measurements. The circuit is dc-biased below the gap and the current through the junction is measured as a function of the frequency of an incoming microwave signal. We observe sharp resonances that we identify to the modes coupled to the junction.

In table S1, we report the measured resonance frequencies as well as the simulated properties of the modes. The mode with the largest impedance probed in the experiment corresponds to the fundamental mode of the quarter wavelength resonator, which is labeled with the index $n = 1$. Because of the reduced length of the second section of the DBR with respect to the Bragg condition, there exists a lower frequency mode, which resonates at 1.9 GHz. This mode has a relatively large impedance, even though it is not fully localized into the last high impedance section of the sample. The modes with even $n = 2, 4, 6$ have a voltage minimum at the position of the junction and therefore a small impedance Z_n as expected for a quarter wavelength resonator. After $n \geq 7$, the mode structure is affected by the DBR and becomes more complicated, we only list the modes that have a significant impedance. Note that the mode impedance as defined in (S.2) is not an intrinsic property of the mode, as in the usual sense of a mode impedance. Here, it depends on the position of the junction and quantifies the vacuum quantum voltage fluctuation as seen by the junction. This definition coincides with the usual one when the junction is located at the maximum of voltage.

Finally, we estimate the Kerr coefficient of the $n = 1$ mode due to the non-linearity of the kinetic inductance. Test wires with the same geometry as the resonator and evaporated in the same conditions exhibit a critical current density of approximately 1 mA µm^{-2}. Following [2], we estimate from this measurement that the Kerr coefficient is approximately $2\pi \times 200$ Hz, which is negligible.

	$\omega_{n,\text{meas.}}/2\pi$ [GHz]	$\omega_{n,\text{sim.}}/2\pi$ [GHz]	Z_n [kΩ]	λ_n
n = 0	1.90	1.89	3.28	0.64
n = 1	5.91	5.91	4.48	0.74
n = 2	12.14	12.19	0.017	0.05
n = 3	15.83	15.54	1.66	0.45
n = 4	23.15	23.12	2×10^{-3}	0.01
n = 5		25.34	1	0.35
n = 6		34.06	$< 1 \times 10^{-3}$	< 0.01
n = 7		35.09	0.71	0.3
n = 8		44.34	0.58	0.27
n = 10		53.80	0.54	0.26
n = 12		63.51	0.35	0.2
n = 14		73.09	0.3	0.19
n = 16		82.67	0.26	0.17

TABLE S1: Resonance frequency, characteristic impedance and displacement amplitude (see main text) of the resonator modes up to 90 GHz. The second column corresponds to measured values, while the others are the results of numerical simulations. The values of ω and Z_n for $n \geq 12$ are obtained by extrapolation.

II. SAMPLE FABRICATION

The sample is micro-fabricated on a 300 µm thick, thermally oxidized, silicon substrate through the following steps.

Granular Aluminum resonator and DBR structure patterning

The design is written on a 1.3 µm thick trilayer resist stack (PMMA 495/PMMA 950/PMMA 950) using electronic beam lithography. After revelation, 10 nm of grAl are deposited by e-beam evaporation at 0° angle (fig. S2a). The oxygen pressure during the evaporation is adjusted to 1.1×10^{-5} mbar and the evaporation rate is 1.5 Å/s. In a following evaporation, without exposing the sample to air, we deposit 30 nm of pure Al with an angle of 45° in the direction perpendicular to the one of the wires (fig. S2b). Because of the the resist thickness and the evaporation angle, only regions with a width exceeding 1.3 µm are covered during this second evaporation. This corresponds, from left to right, to the measurement line, the second DBR section and a final 2.5x2.5 µm^2 square contact that is used to connect the junction.

In a second step, we pattern the remaining part of the 50 Ω measurement line and a central ground plane, which is used to connect the other side of the junction (fig. S2c). These structures are realized through optical lithography

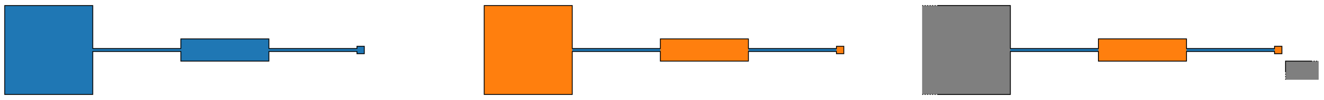

(a) Evaporation of 10 nm of grAl at 0° angle.

(b) Evaporation of 30 nm of Al at 45° angle.

(c) Patterning of measurement lines and ground contacts in Al.

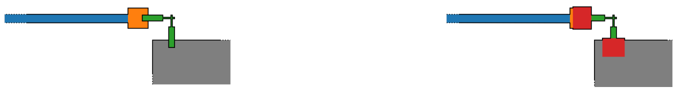

(d) Realization of the Al/AlOx/Al tunnel junction.

(e) Connection of the junction contacts.

FIG. S2: Main steps of the fabrication process.

and liftoff of a 60 nm thick Al layer. In order to obtain a good dc contact with the previously deposited measurement line, an argon ion beam etching step is used before the evaporation. At the end of the process a 30 nm layer of Nb is deposited at the back of the sample for the ground plane.

Tunnel junction patterning

The Al/AlOx/Al junction is realized through a standard double angle evaporation using a PMMA bilayer resist without any suspended bridge ("Manhattan technique“, see fig. S2d). The width of the junction arms is 150 nm and the oxidation is performed at 10 mbar oxygen pressure (static) for 20 minutes. In a final step, we connect the junction contacts to both the resonator and the ground plane using a last step of optical lithography, followed by Ar ion beam etching and the evaporation of 80 nm of Al (fig. S2e).

III. EXPERIMENTAL SETUP

The sample is glued on a gold-plated copper sample holder and bonded to a microwave PCB. The sample holder is fixed on the 10 mK stage of a Cryoconcept dry dilution refrigerator.

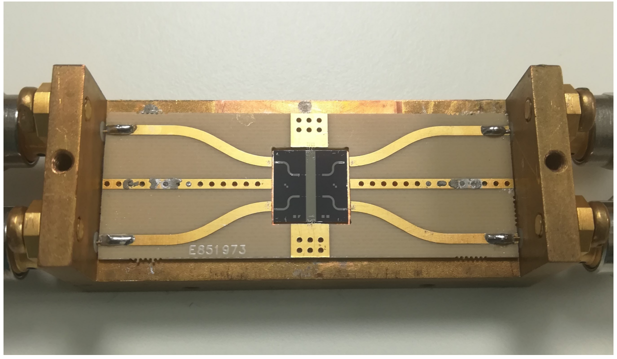

FIG. S3: Picture of the sample holder and of the PCB to which the sample is wire bonded.

The experimental setup used to measure the sample is shown in figure S4. All the microwave measurements presented in the manuscript are performed with a vector network analyzer connected to the RF input and output ports. The microwave drive is sent to the sample through an attenuated coaxial line and its reflection is collected by a different line using a circulator. The reflected signal is amplified by a cryogenic HEMT amplifier at 4 K followed by two room temperature amplifiers. In order to dc-bias the junction, we use a voltage divider followed by a filtering capacitor and a bias tee. A 10 kΩ resistance is mounted in series with the sample to measure the current through the junction.

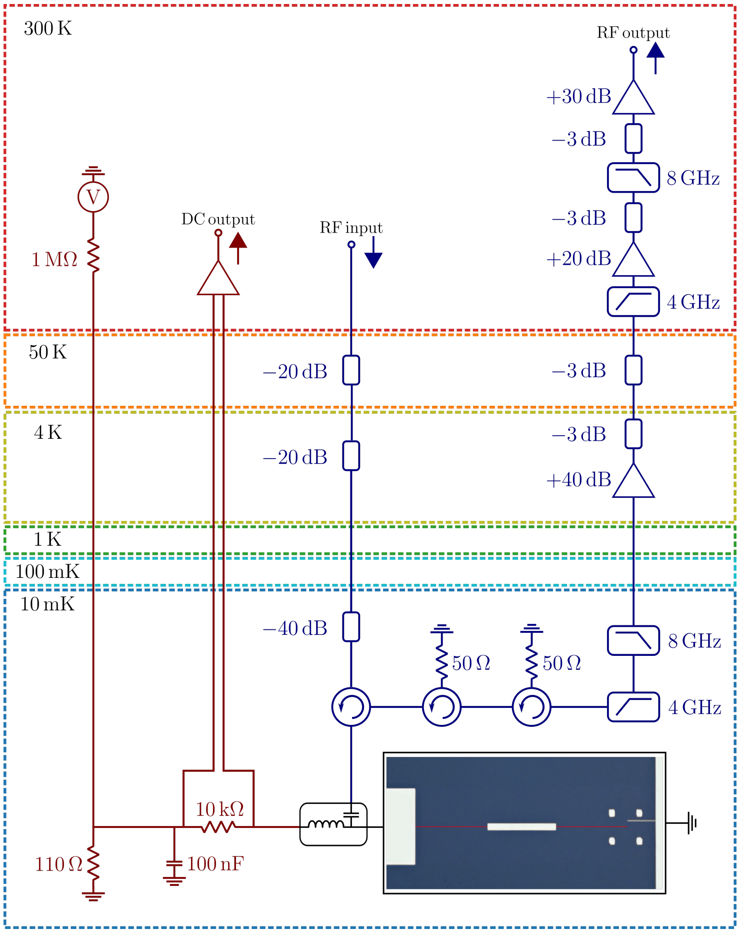

FIG. S4: Microwave and dc setup used for the experiment.

IV. JUNCTION ADMITTANCE

When the impedance of the mode coupled to the junction is small compared to the quantum of resistance ($\lambda \ll 1$), the resonator field can be treated classically. In this case, the effect of charge tunneling can be modeled by treating the junction as a frequency and voltage dependent admittance Y_J [3]. This admittance also varies with the microwave amplitude seen by the junction. Supposing a weak microwave amplitude, the real and imaginary parts of the admittance can be expanded to first order in the microwave amplitude as [3, 4]:

$$\mathrm{Re}(Y_J) = \frac{e}{2\hbar\omega}\left[I(V+\hbar\omega/e) - I(V-\hbar\omega/e)\right] \tag{S.3}$$

$$\mathrm{Im}(Y_J) = \frac{e}{2\hbar\omega}\left[I^{\mathrm{KK}}(V+\hbar\omega/e) + I^{\mathrm{KK}}(V-\hbar\omega/e) - 2I^{\mathrm{KK}}(V)\right] . \tag{S.4}$$

Here, I is the quasiparticle current that is defined as

$$I(V) = \frac{1}{R_T}\int_{-\infty}^{\infty} n_L(V')n_R(V'-V)[f(V'-V) - f(V')]\, dV' , \tag{S.5}$$

where n_R (n_L) is the dimensionless density of states in the right (left) contact, which tend to one at voltages far below and far above the gap, and $f(V)$ is the Fermi distribution. Finally, $I^{\mathrm{KK}}(V)$ is the Kramers-Kronig transform of $I(V)$ as defined in the main text. Their variations near the superconducting gap $V = 2\Delta/e$ are shown in figure S5.

Taking into account this admittance in the calculation of the resonator mode properties, the real part of the admittance translates into a loss rate κ and the imaginary part into a resonance frequency shift $\delta\omega$. The two

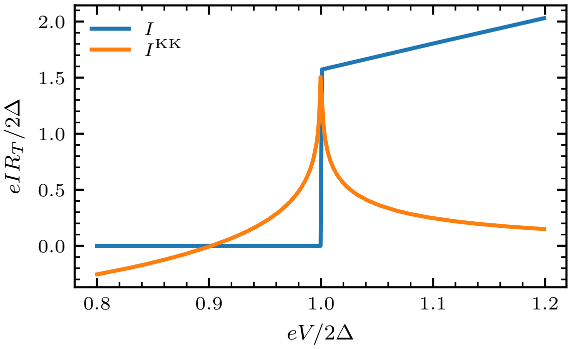

FIG. S5: Quasiparticle current and its Kramers-Kronig transform near the superconducting gap.

quantities are given by

$$\kappa = \frac{\lambda^2}{e}\left[I(V+\hbar\omega/e) - I(V-\hbar\omega/e)\right] \tag{S.6}$$

$$\delta\omega = -\frac{\lambda^2}{2e}\left[I^{\mathrm{KK}}(V+\hbar\omega/e) + I^{\mathrm{KK}}(V-\hbar\omega/e) - 2I^{\mathrm{KK}}(V)\right] . \tag{S.7}$$

These expressions can be derived using equations (2) and (3) of the main text in the limit of small λ. In a quantum approach, they correspond to the usual single photon loss and frequency shift that arise when an harmonic oscillator mode is linearly coupled to a bath. In the strong impedance regime, where $\lambda \approx 1$, this approach is not valid, and a quantum treatment of the field, and in particular of the displacement operator appearing in the tunnel Hamiltonian, is necessary.

V. QUANTUM MASTER EQUATION

In order to describe the quantum dynamics of the resonator field in the high impedance regime, we use a master equation formalism and treat the junction as a bath and the resonator as an open quantum system. Following the standard quantum optics approach based on the Born-Markov and on the secular approximation [5], one obtains a master equation in the Lindblad form for the density matrix of the resonator modes as detailed, for example, in [6]. The master equation is written in terms of jump operators $\hat{A}_l$ operators that come from the expansion of the displacement operator $\exp[i\lambda(\hat{a}+\hat{a}^\dagger)]$ in the Fock state basis. Their effect is to create l photons when $l > 0$ and annihilate $-l$ photons when $l < 0$. They are defined, for positives l, as

$$\hat{A}_{l>0} = \sum_{n=0}^{\infty} |n+l\rangle \langle n+l|\, e^{i\lambda(\hat{a}+\hat{a}^\dagger)}\, |n\rangle \langle n| \, . \tag{S.8}$$

The operators with $l < 0$ are defined through $\hat{A}_{-l} = (-1)^l \hat{A}_l^\dagger$. For $l \geq 0$, the matrix elements of $\hat{A}_l$ can be obtained from the following identity

$$\langle n+l|e^{i\lambda(\hat{a}+\hat{a}^\dagger)}|n\rangle = \sqrt{\frac{n!}{(n+l)!}}\, e^{-\lambda^2/2}(i\lambda)^l L_n^{(l)}(\lambda^2)\, , \tag{S.9}$$

where $L_n^{(l)}$ are the generalized Laguerre polynomials. In the main text, we define $\alpha_{nl} = |\langle n+l|e^{i\lambda(\hat{a}+\hat{a}^\dagger)}|n\rangle|^2$, which can be rewritten in terms of $\hat{A}_l$ operators as $\alpha_{nl} = |\langle n+l|A_l|n\rangle|^2$ for $l \geq 0$. When the non-linearity is strong ($\lambda \approx 1$), quantum jumps, generated by the $\hat{A}_l^\dagger$ operators, lead to transitions from $|n\rangle$ to $|n-l\rangle$. This is in stark contrast to the usual quantum optics situation, where the two possible jump operators are $\hat{a}$ and $\hat{a}^\dagger$, which only couples adjacent Fock states. In the case where many modes are coupled to the junction, jump operators can be defined for each mode using the value of λ obtained from numerical simulations of the mode impedance seen by the junction (see I). In order to obtain more compact expressions, we define

$$\hat{A}_{l_0,l_1,\ldots} = \hat{A}_{l_0} \otimes \hat{A}_{l_1} \otimes \ldots \, , \tag{S.10}$$

where $\hat{A}_{l_n}$ is the operator defined in (S.8) associated to mode n. The other ingredients entering the master equation are the real and imaginary part of the single sided Fourier transform of the bath correlation function. In the case of a tunnel junction, one obtains

$$\gamma(V) = \frac{1}{eR_T}\int_{-\infty}^{\infty} n_R(V')n_L(V+V')f(V')(1-f(V+V'))\,dV' \tag{S.11}$$

$$\epsilon(V) = \frac{1}{2\pi}\mathcal{P}\int_{-\infty}^{\infty}\frac{\gamma(V')}{V-V'}\,dV'\,. \tag{S.12}$$

With these definitions, the master equation for the density matrix of the modes is

$$\frac{d\rho}{dt} = -i\Big[\hat{H}_{\rm LS},\rho\Big] + \sum_{l_0,l_1,\ldots=-\infty}^{\infty}\left[\gamma(V+\sum_n l_n\hbar\omega_n/e)+\gamma(-V+\sum_n l_n\hbar\omega_n/e)\right]\mathcal{D}[\hat{A}^\dagger_{l_0,l_1,\ldots}](\rho) \tag{S.13}$$

$$\hat{H}_{\rm LS} = \sum_{l_0,l_1,\ldots=-\infty}^{\infty}\left[\epsilon(V+\sum_n l_n\hbar\omega_n/e)+\epsilon(-V+\sum_n l_n\hbar\omega_n/e)\right]\hat{A}_{l_0,l_1,\ldots}\hat{A}^\dagger_{l_0,l_1,\ldots} \tag{S.14}$$

$$\mathcal{D}[\hat{X}](\rho) = \hat{X}\rho\hat{X}^\dagger - \frac{1}{2}\Big\{\hat{X}^\dagger\hat{X},\rho\Big\}\,. \tag{S.15}$$

The first term of equation (S.13) describes the Lamb shift of the resonator energy levels with associated Hamiltonian $H_{\rm LS}$, while the second term corresponds to photo-assisted tunneling events, during which one electron tunnels and photons are absorbed and/or created in the resonator modes. As required by gauge invariance, the master equation is unchanged by $V\to -V$. While deriving this equation, we assume that the mode frequencies are not commensurate when performing the secular approximation. The bath functions $\gamma(V)$ and $\epsilon(V)$ can be related to the $I(V)$ characteristic of the junction by noticing that

$$I(V) = e[\gamma(V)-\gamma(-V)] \quad\approx e\,\gamma(V) \tag{S.16}$$

$$I^{\rm KK}(V) = -2e[\epsilon(V)+\epsilon(-V)] \approx -2e\,\epsilon(V)\,. \tag{S.17}$$

The last approximations are valid for positive bias and low temperature. With these approximations, the master equation can be rewritten as

$$\frac{d\rho}{dt} = -i\Big[\hat{H}_{\rm LS},\rho\Big] + \frac{1}{e}\sum_{l_0,l_1,\ldots=-\infty}^{\infty} I(V+\sum_n l_n\hbar\omega_n/e)\mathcal{D}[\hat{A}^\dagger_{l_0,l_1,\ldots}](\rho) \tag{S.18}$$

$$\hat{H}_{\rm LS} = -\frac{1}{2e}\sum_{l_0,l_1,\ldots=-\infty}^{\infty} I^{\rm KK}(V+\sum_n l_n\hbar\omega_n/e)\hat{A}_{l_0,l_1,\ldots}\hat{A}^\dagger_{l_0,l_1,\ldots}\,. \tag{S.19}$$

This master equation is only valid for positive bias and as long as the energy shift $\sum_n l_n\hbar\omega_n$ remains small compared to the gap 2Δ, which is the case in our experiment. This multimode master equation is in general difficult to solve. In the quantum Zeno experiment presented in the main text, only the 6 GHz mode is pumped. We therefore assume that the other modes do not participate to the dynamics and remain in vacuum. When tracing out these modes, we have to compute

$$\begin{aligned}
{\rm Tr}_{0,2,\ldots}\left[\hat{A}_{l_0,l_1,\ldots}\hat{A}^\dagger_{l_0,l_1,\ldots}\rho\right] &= \langle 0|\hat{A}_{l_0}\hat{A}^\dagger_{l_0}|0\rangle\ \langle 0|\hat{A}_{l_2}\hat{A}^\dagger_{l_2}|0\rangle\ldots\hat{A}_{l_1}\hat{A}^\dagger_{l_1}\rho_1 &\text{(S.20)}\\
&= |\,\langle 0|\hat{A}_{l_0}|{-l_0}\rangle\,|^2\,|\,\langle 0|\hat{A}_{l_2}|{-l_2}\rangle\,|^2\ldots\hat{A}_{l_1}\hat{A}^\dagger_{l_1}\rho_1 &\text{(S.21)}\\
&= \begin{cases} W_{l_0}e^{-\lambda_0^2}\,W_{l_2}e^{-\lambda_2^2}\ldots\hat{A}_{l_1}\hat{A}^\dagger_{l_1}\rho_1 & \text{if } l_0\le 0,\ l_2\le 0,\ldots\\ 0 \ \ \text{otherwise}\,, \end{cases} &\text{(S.22)}
\end{aligned}$$

with $W_{l_n} = \lambda_n^{-2l_n}/(-l_n)!$. The master equation for the $n=1$ mode is then given by

$$\frac{d\rho_1}{dt} = -i\Big[\hat{H}_{\rm LS},\rho_1\Big] + \frac{1}{e}\sum_{l_1=-\infty}^{\infty}\ \sum_{l_0,l_2,\ldots=-\infty}^{0} W_{l_0}W_{l_2}\ldots\tilde{I}(V+\sum_n l_n\hbar\omega_n/e)\mathcal{D}[\hat{A}^\dagger_{l_1}](\rho_1) \tag{S.23}$$

$$\hat{H}_{\rm LS} = -\frac{1}{2e}\sum_{l_1=-\infty}^{\infty}\ \sum_{l_0,l_2,\ldots=-\infty}^{0} W_{l_0}W_{l_2}\ldots\tilde{I}^{\rm KK}(V+\sum_n l_n\hbar\omega_n/e)\hat{A}_{l_1}\hat{A}^\dagger_{l_1}\,, \tag{S.24}$$

The modified current characteristic $\tilde{I}$ takes into account the dynamical Coulomb blockade (DCB) due to the other modes. It is defined as

$$\tilde{I}(V) = e^{-\lambda_0^2} e^{-\lambda_2^2} e^{-\lambda_3^2} \dots I(V)\,. \tag{S.25}$$

The multi-mode DCB factor is the product of the blockade factors $|\langle 0|\exp\big[i\lambda_n(\hat{a}_n + \hat{a}_n^\dagger)\big]|0\rangle|^2 = e^{-\lambda_n^2}$ for each mode, except the 6 GHz mode. As mentioned in the main text, this is equivalent to an increase of the tunnel resistance by a factor $\Pi_{n\neq 1} e^{\lambda_n^2}$.

The master equation can be further simplified by keeping only the $l_n = 0$ term in the sum over the other modes. This assumes that the effect of these modes is simply to renormalize the tunnel resistance. We then obtain

$$\frac{d\rho_1}{dt} = -i\Big[\hat{H}_{\mathrm{LS}}, \rho_1\Big] + \frac{1}{e}\sum_{l_1=-\infty}^{\infty} \tilde{I}(V + l_1\hbar\omega_1/e)\mathcal{D}[\hat{A}_{l_1}^\dagger](\rho_1) \tag{S.26}$$

$$\hat{H}_{\mathrm{LS}} = -\frac{1}{2e}\sum_{l_1=-\infty}^{\infty} \tilde{I}^{\mathrm{KK}}(V + l_1\hbar\omega_1/e)\hat{A}_{l_1}\hat{A}_{l_1}^\dagger\,, \tag{S.27}$$

For voltages below the gap, the simplification of the jump terms only neglects the contribution of the 1.9 GHz mode. In particular, we neglect processes where photons in the 6 GHz are converted into photons at 1.9 GHz and the remaining energy is taken by the tunneling electron (see figure S6). The higher frequency modes have no influence because of energy conservation. Computing the loss rate for state $|n\rangle$ using (S.26) leads to equation (2) of the main text. The expression of $\hat{H}_{\mathrm{LS}}$ is more affected than the jump terms, because all the modes contribute as discussed in more details below (see VII).

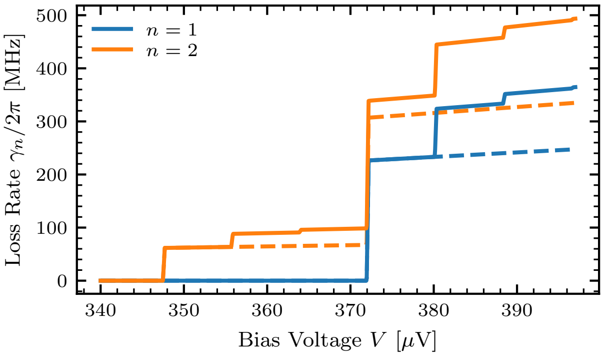

FIG. S6: Comparison of the different predictions for the loss rates of the first two Fock states $n = 1$ and $n = 2$ as a function of voltage. The dashed lines correspond to predictions of (S.26), as plotted in figure 2a of the main text. Solid lines correspond to the predictions of (S.23).

VI. ZENO DYNAMICS AND PUMP CALIBRATION

We now use the previously derived master equation to explain the quantum Zeno dynamics observed in the experiment. Quantum Zeno dynamics happens when we dc-bias the junction at 363 µV, which lies in the voltage region where the $l \geq 2$ processes are the dominant loss process. We measure the resonator reflected spectrum S_{11} for different microwave powers (see inset of figure 3b of the main text) and extract the mode amplitude from these measurements. This requires to know the pump amplitude, which is defined as $\eta = \sqrt{\kappa_c P/\hbar\omega}$, where κ_c is the coupling loss rate and P is the incoming microwave power on the sample. The coupling κ_c is measured independently. The incoming power P depends on the total attenuation of the experimental setup that needs to be calibrated. We do so by measuring the resonance fwhm and by comparing it to the one of a two level system. In a two level system, the fwhm is equal to $\sqrt{\kappa^2 + 4\Gamma^2}$, with κ being the total loss rate and Γ the power broadening, which varies with the pump amplitude η as $\Gamma = \sqrt{2}\eta$. By assuming the same power dependence on the measured Γ, we arrive at the attenuation of the line that pumps the resonator.

We extract the loss rate κ and the broadening Γ by fitting the spectra measured at different power using the two-level formula [7]

$$S_{11} = 1 - \kappa_c \frac{\kappa/2 - i\delta}{(\kappa/2)^2 + \delta^2 + \Gamma^2}\,, \tag{S.28}$$

where δ is the frequency detuning between the pump and the $0 \to 1$ resonance. In figure S7, we plot the spectrum measured at $P = -162$ dBm together with the fitted S_{11} (red curve). This procedure is repeated for every power, and by fitting the slope of Γ with power, we obtain the attenuation and the value of η as used in figure 3 of the main text. Once the pump is calibrated, we extract the resonator field intensity from the measured spectra through

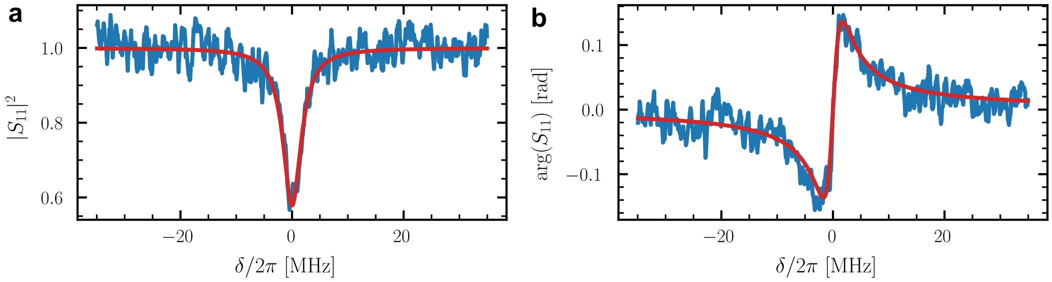

FIG. S7: **a** Squared modulus and **b** phase of the reflection spectrum measured at $P = -162$ dBm. The red curve shows the fitted spectrum assuming a two-level system model.

$$\langle \hat{a} \rangle = \frac{\eta}{\kappa_c}\,(1 - S_{11})\,. \tag{S.29}$$

A plot of $|\langle \hat{a} \rangle|^2$ at resonance is shown in figure 3a of the main text. We compare this measured intensity to the predictions of the following master equation

$$\frac{d\rho}{dt} = -i\left[\hat{H}, \rho\right] + \kappa \mathcal{D}[\hat{a}](\rho) + \frac{1}{e}\sum_{l=-\infty}^{\infty} \tilde{I}(V + l\hbar\omega/e)\mathcal{D}[\hat{A}_l^\dagger](\rho) \tag{S.30}$$

$$\hat{H} = i\eta(\hat{a} - \hat{a}^\dagger) - \delta\,\hat{a}^\dagger\hat{a} + \hat{H}_{\mathrm{LS}} \tag{S.31}$$

$$\hat{H}_{\mathrm{LS}} = -\frac{1}{2e}\sum_{l=-\infty}^{\infty} \tilde{I}^{\mathrm{KK}}(V + l\hbar\omega/e)\hat{A}_l\hat{A}_l^\dagger\,. \tag{S.32}$$

This master equation is the simplified single mode master equation obtained in (S.26), where we add a pump term with amplitude η, a detuning term proportional to δ, and also include a single photon loss rate κ in order to take into account single photon loss from various origins. This loss rate is $\kappa = 2\pi \times 4.2$ MHz ($\kappa = 2\pi \times 2.7$ MHz) for $V = 363$ μV ($V = 284$ μV). These values are chosen to reproduce the observed resonance width at low pump power. The expectation value of $\hat{a}$ is then obtained from the steady state solution of the master equation (calculated using the QuTip Python software [8]) and allows us to plot the curve in figure 3a of the main text. We also compute the expected S_{11} and apply the same fitting procedure as for the experimental data and obtain the theoretical power broadening Γ (shown in figure 3b of the main text) and the loss rate κ (plotted in figure S8).

In figure S9, we compare the results of simulations including or not the Lamb shift term H_{LS}. The non-linearity introduced by the Lamb shift significantly contributes to the blockade of the $1 \to 2$ transition in addition to the Zeno effect. The figure of merit of the observed blockade can be quantified by the range of pump amplitude over which the mode behaves as a saturated two level system. Supposing that the mode is subject only to one and two photon loss, without any non-linearity, this range is set by the ratio $\tilde{\gamma}_2/\tilde{\gamma}_1$, where $\tilde{\gamma}_n$ is the effective loss rate of state $|n\rangle$ in this model. We find that, without Lamb shift, the ratio $\tilde{\gamma}_2/\tilde{\gamma}_1$ is about 15, with $\tilde{\gamma}_2$ being $2\pi \times 65$ MHz and $\tilde{\gamma}_1 = 2\pi \times 4.2$ MHz. When we include the Lamb shift $\tilde{\gamma}_2$ increases approximately to $2\pi \times 100$ MHz with the ratio $\tilde{\gamma}_2/\tilde{\gamma}_1$ being about 25.

Finally, we simulate a master equation modeling ideal Zeno dynamics (see figure S9). We suppose that the Hamiltonian is given by equation (S.31) without the Lamb shift term. We then include two jump operators. The first one describes single photon loss with rate κ and the second one is the projector $|0\rangle\langle 0| + |1\rangle\langle 1|$, which measures if the state is in the $\{|0\rangle, |1\rangle\}$ subspace. The projection rate γ_P is adjusted to fit the data in the region $\eta < 2\pi \times 3$ MHz, we obtain $\gamma_P = 2\pi \times 215$ MHz, which is comparable to the rate of $2\pi \times 100$ MHz found above.

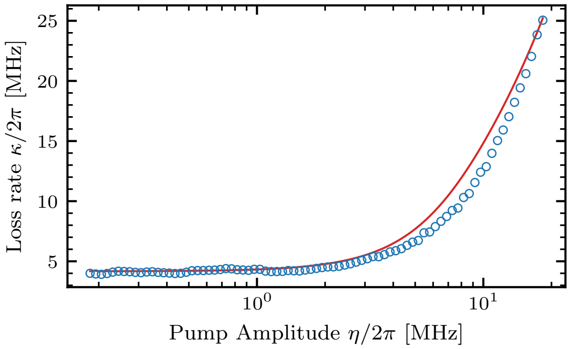

FIG. S8: Comparison between the measured (blue data) and simulated (red curve) evolution of the loss rate κ. The curves show that κ remains constant, as in a two level system, as long as the Zeno effect limits the dynamics of the system to the first two Fock states. When the Zeno dynamics break down, the loss rate increases quadratically with the pump amplitude.

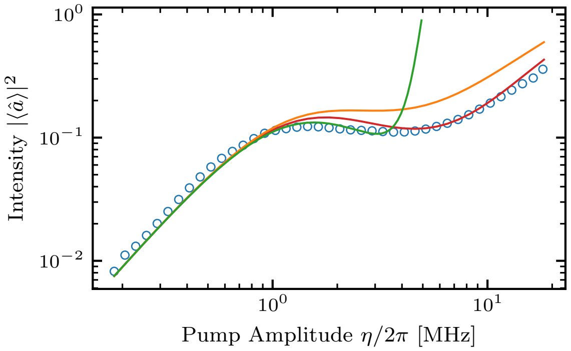

FIG. S9: Comparison of the mode intensity predicted by different master equations. The red (orange) curve corresponds to the solution of (S.30) with (without) the Lamb shift term at $V = 363$ µV. The green curve shows the prediction for an ideal projective measurement in the $\{|0\rangle, |1\rangle\}$ subspace. The blue points correspond to the experimental results shown in figure 3 of the main text.

VII. LAMB SHIFT

From the expression of $\hat{H}_{\mathrm{LS}}$ derived in V, the Lamb shift $\delta\omega_n$ of the $|n\rangle$ state of the 6 GHz mode, with all the other modes in vacuum, is given by

$$\delta\omega_n = -\frac{1}{2e} \sum_{l_1=-\infty}^{\infty} \sum_{l_0, l_2, \ldots = -\infty}^{0} W_{l_0} W_{l_2} \ldots \tilde{I}^{\mathrm{KK}}(V + \sum_n l_n \hbar\omega_n / e) \langle n | \hat{A}_{l_1} \hat{A}_{l_1}^{\dagger} | n \rangle \ . \tag{S.33}$$

As discussed above, the main effect of the other modes is to renormalize the tunnel resistance, but the remaining terms modify the V dependence beyond a simple renormalization of R_T. Including all the modes presented in I, we use this expression to compute the shift of the $|0\rangle$, $|1\rangle$ and $|2\rangle$ states, from which we deduce the curves plotted in figure 4 of the main text. As previously discussed in V, a natural approximation consists in keeping only the $l_n = 0$ terms in the sum, which leads to

$$\delta\omega_n = -\frac{1}{2e} \sum_{l=-\infty}^{\infty} \tilde{I}^{\mathrm{KK}}(V + l\hbar\omega/e) \langle n | \hat{A}_l \hat{A}_l^{\dagger} | n \rangle \ . \tag{S.34}$$

We have dropped the mode index and the A_l operator implicitly acts on the 6 GHz mode. By using the identities $\langle n | \hat{A}_l \hat{A}_l^{\dagger} | n \rangle = |\langle n | \hat{A}_l | n - l \rangle|^2$ if $n \geq l \geq 0$ and $\langle n | \hat{A}_l \hat{A}_l^{\dagger} | n \rangle = |\langle n - l | \hat{A}_{-l} | n \rangle|^2$ if $l \leq 0$, we obtain

$$\delta\omega_n = -\frac{1}{2e} \left[\sum_{l=1}^{n} \tilde{I}^{\mathrm{KK}}(V + l\hbar\omega/e) \alpha_{n-l,l} + \sum_{l=0}^{\infty} \tilde{I}^{\mathrm{KK}}(V - l\hbar\omega/e) \alpha_{n,l} \right] , \tag{S.35}$$

which corresponds to equation (3) of the main text. From the definition of the generalized Laguerre polynomials $L_0^{(l)}(\lambda^2) = 1$ and $L_1^{(l)}(\lambda^2) = 1 + l - \lambda^2$, we arrive at the following expression for the shift of the $0 \to 1$ transition

$$\delta\omega_{01} = -\frac{\lambda^2 e^{-\lambda^2}}{2e} \sum_{l=0}^{\infty} \frac{\lambda^{2l}}{l!} \left[\tilde{I}^{\mathrm{KK}}(V - (l+1)\hbar\omega/e) + \tilde{I}^{\mathrm{KK}}(V - (l-1)\hbar\omega/e) - 2\tilde{I}^{\mathrm{KK}}(V - l\hbar\omega/e) \right], \tag{S.36}$$

which corresponds to equation (4) of the main text. Keeping only the $l = 0$ term, we obtain a semi-classical expression, which is equivalent to the prediction of a classical model where the tunnel resistance is renormalized as $R_T \to R_T \Pi_n e^{\lambda_n^2}$.

We compare these different approximations and the exact expression in to our data in figure S10. When we compare our data to the theoretical predictions, as here or as in figure 4 of the main text, we allow for a small frequency shift of the transition frequency (vertical offset) and also for a small shift of the gap (horizontal offset). With these adjustments, our data are in better agreement with the exact multi-mode calculation (S.33) than with the simplified formula obtained in (S.36).

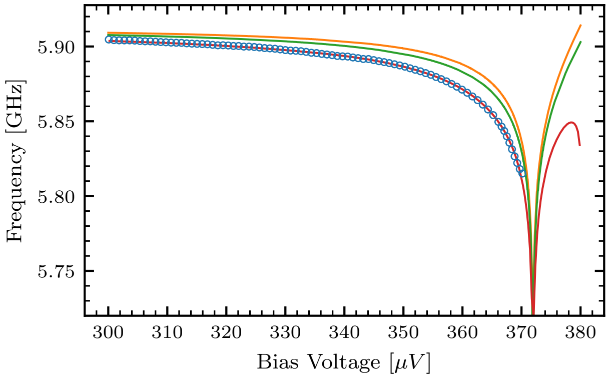

FIG. S10: Comparison of the different predictions for the shift $\delta\omega_{01}$ of the fundamental resonance together with the experimental data (blue circles). The red curve corresponds to the multi-mode prediction (S.33), the green curve to the single mode approximation (S.36) and the orange curve to the semi-classical model, which is obtained by keeping only the $l = 0$ term in (S.36).

[1] S. M. Girvin, *Circuit QED: Superconducting Qubits Coupled to Microwave Photons* (Oxford University Press, 2014).
[2] N. Maleeva, L. Grünhaupt, T. Klein, F. Levy-Bertrand, O. Dupre, M. Calvo, F. Valenti, P. Winkel, F. Friedrich, W. Wernsdorfer, A. V. Ustinov, H. Rotzinger, A. Monfardini, M. V. Fistul, and I. M. Pop, Nature Communications **9**, 1 (2018), arXiv:1802.01859.
[3] J. R. Tucker and M. J. Feldman, Reviews of Modern Physics **57**, 1055 (1985).
[4] A. H. Worsham, N. G. Ugras, D. Winkler, D. E. Prober, N. R. Erickson, and P. F. Goldsmith, Physical Review Letters **67**, 3034 (1991).
[5] H.-P. Breuer and F. Petruccione, *The Theory of Open Quantum Systems* (Oxford University Press, 2007).
[6] J. Estève, M. Aprili, and J. Gabelli, arXiv:1807.02364 (2018).
[7] C. Cohen-Tannoudji, J. Dupont-Roc, and G. Grynberg, *Atom—Photon Interactions* (Wiley, 1998).
[8] J. Johansson, P. Nation, and F. Nori, Computer Physics Communications **184**, 1234 (2013).
